# FOLFOX6 and bevacizumab in non-optimally resectable liver metastases from colorectal cancer

**DOI:** 10.1038/bjc.2011.43

**Published:** 2011-03-08

**Authors:** F Bertolini, N Malavasi, L Scarabelli, F Fiocchi, B Bagni, C Del Giovane, G Colucci, G E Gerunda, R Depenni, S Zironi, A Fontana, E Pettorelli, G Luppi, P F Conte

**Affiliations:** 1 Oncology, Haematology and Respiratory Diseases Department, University Hospital of Modena, Modena, Italy; 2 Radiology Institute, University Hospital of Modena, Modena, Italy; 3 Nuclear Medicine Division, University Hospital of Modena, Modena, Italy; 4 General Surgery, Vignola Hospital, Azienda USL Modena, Modena, Italy; 5 Multiorgan Transplantation Division, University Hospital of Modena, Modena, Italy

**Keywords:** bevacizumab, liver metastases, colorectal cancer

## Abstract

**Background:**

In patients with colorectal liver metastases (CLM) R0 resection significantly improves overall survival (OS).

**Methods:**

In this report, we present the results of a phase II trial of FOLFOX6+bevacizumab in patients with non-optimally resectable CLM. Patients received six cycles of FOLFOX6+ five of bevacizumab. Patients not achieving resectability received six additional cycles of each. A PET-CT was performed at baseline and again within 1 month after initiating treatment.

**Results:**

From September 2005 to July 2009, 21 patients were enrolled (Male/Female: 15/6; median age: 65 years). An objective response (OR) was documented in 12 cases (57.1% complete responses (CRs): 3, partial response (PR): 9); one patient died from toxicity before surgery. Thirteen patients underwent radical surgery (61.9%). Three (23%) had a pathological CR (pCR). Six patients (46.1%) experienced minor postsurgical complications. After a median 38.8-month follow-up, the median OS was 22.5 months. Patients achieving at least 1 unit reduction in Standard uptake value (SUV)max on PET-CT had longer progression-free survival (PFS) (median PFS: 22 *vs* 14 months, *P*=0.001).

**Conclusions:**

FOLFOX6+bevacizumab does not increase postsurgical complications, yields high rates of resectability and pCR. Early changes in PET-CT seem to be predictive of longer PFS.

Resection of metastases is the optimal treatment goal in metastatic colorectal cancer. In particular, approximately 50% of patients with colorectal cancer will develop metastases (synchronous or metachronus) during their clinical history and 30–35% will have liver metastases only. Only 10–20% of patients are candidates for surgery, whereas the remainder are candidates for only palliative treatment. In resectable patients, the cure rate is about 20–30%, with a survival rate of 40%, but 70–80% will relapse within 2 years (Chua and Cunningham, 2006; Van Cutsem *et al*, 2006).

Surgery may lead to long-term survival: on the basis of the Oncosurge international registry of patients operated on for colorectal liver metastasis (http://www.livermetsurvey.org, 2009), the 5- and 10-year post-hepatectomy survival rates are 40 and 25%, respectively.

Adam *et al* reported a 13% rate of conversion to resectability for patients with unresectable disease after tumour downsizing by chemotherapy, associated with a 5-year survival rate of 33% after hepatectomy. Moreover, the 10-year long-term survival of liver resection for primarily resectable patients is 30 *vs* 23% for patients with initially unresectable metastases that become resectable after chemotherapy (Adam *et al*, 2004).

Although there is consensus on the role of surgery in CLM, the best neoadjuvant chemotherapy is still not clear. In general, chemotherapy should aim to achieve a higher response rate, to be clinically manageable and to have minimal toxicity.

There is a strong correlation between response rates and the resection rate, both in patients selected for liver metastases only, and in non-selected patients; however, patient selection and efficacy of neoadjuvant chemotherapy are both strong predictors for resectability of CLM (Folprecht *et al*, 2005).

Numerous combination therapies have been tested in this setting: doublets, doublets plus biologics, triplets and triplets plus biologics (early results), with a response rate of 34–80% and a resectability rate of 2–92% (depending on patient selection) (Tournigand *et al*, 2004; Falcone *et al*, 2007; Gruenberger *et al*, 2008; Bokemeyer *et al*, 2009; Okines *et al*, 2009; Van Cutsem *et al*, 2009; Wong *et al*, 2009; Folprecht *et al* 2010; Masi *et al*, 2010).

Moreover, when the present study was planned, the use of bevacizumab in the neoadjuvant setting was limited by the potential for mechanism of action-based increase of postoperative morbidity from haemorrhagic complications and delay of wound healing.

Therefore, we designed this phase II trial to estimate the percentage of patients amenable to complete resection after 3 months of neoadjuvant combination therapy with FOLFOX6+bevacizumab.

## Patients And Methods

This phase II study was designed and conducted according to the International Conference on Harmonisation Harmonized Tripartite Guideline for Good Clinical Practice (http://www.ifpma.org/pdfifpma/e6.pdf). The protocol was approved by the Local Ethics Committee of the Province of Modena, Italy.

### Eligibility criteria

Eligible patients were aged ⩾18 years with a histologically or cytologically proven diagnosis of colorectal cancer with non-optimally resectable liver metastases that are evaluable according to RECIST criteria and not previously treated with chemotherapy for metastatic disease. Non-optimally resectable was defined as: single nodule (>5 cm); multiple nodules (>4 cm) and/or bilobar lesions; synchronous liver metastases (especially for patients with rectal cancer). Moreover, patients had to have an Eastern Cooperative Oncology Group Performance Status of 0–1 and adequate organ function: neutrophils ⩾1.5 × 10^9^ l^−1^, platelets ⩾100 × 10^9^ l^−1^, and haemoglobin ⩾9 g dl^−1^, bilirubin level of normal or <1.5 × upper limit of normal (ULN) of the Institutional normal values, aspartate aminotransferase and/or alanine aminotransferase ⩽5 × ULN, alkaline phosphatase, ⩽5 × ULN, serum creatinine <1.5 × ULN, urine dipstick of proteinuria <2+ (patients with proteinuria on baseline dipstick urinalysis ⩾2+ had to undergo a 24-h urine collection and must have had ⩽1 g of protein per 24 h); signed informed consent was required before beginning protocol-specific procedures. Patients had to have a life expectancy of at least 12 weeks and be accessible for treatment and follow up.

### Exclusion criteria

Patients were excluded for unresectable extra-hepatic disease; evidence of peritoneal carcinosis at baseline; widespread liver involvement (>70–80%); symptomatic and/or unstable pre-existing brain metastases; history of inflammatory bowel disease and/or acute/subacute bowel obstruction; serious non-healing wound or ulcer; evidence of bleeding diathesis or coagulopathy; uncontrolled hypertension; active clinically significant cardiovascular disease, for example, cerebrovascular accidents (⩽6 months), myocardial infarction (⩽6 months), unstable angina, New York Heart Association grade II or greater congestive heart failure, serious cardiac arrhythmia requiring medication; treatment with anticoagulants within the previous 10 days; chronic, daily treatment with high-dose aspirin (>325 mg day^−1^) or other medications known to predispose to gastrointestinal ulceration.

Other exclusion criteria were major surgical procedure, open biopsy or significant traumatic injury in the previous 28 days, or anticipated need for major surgery during the course of the study. Patients with symptomatic peripheral neuropathy ⩾grade 1 according the National Cancer Institute Common Toxicity Criteria (NCI-CTC) were excluded.

### Statistical considerations

Primary end point of the study was the percentage of patients amenable to complete resection after chemotherapy. The sample size was calculated on the basis of the two-stage design by Simon. Assuming that the standard resectability rate for liver metastases was 18%, we planned to detect an increase in the resectability rate of up to 33%. Therefore, a total of 46 patients were needed to provide a power of 80% and an *α*-error equal to 5%. Sixteen patients were planned for the first stage and another 30 patients to be enrolled during the second stage. If three or fewer R0 surgical resections were performed during the first stage, then the trial would be stopped. The study would also be discontinued if we observed a >30% adverse event rate (major wound healing complications, haemorrhage, gastrointestinal perforation, fistula and abscess) after surgery.

Secondary end points were: the incidence of postoperative complications within 60 days of surgery; antitumor activity in terms of overall response rate (complete and PRs); toxicity and tolerability of the combination; time to progression (TTP) and OS. In addition, the predictive value of PET-CT for response to neoadjuvant treatment was investigated.

Overall response was defined as the best response recorded from the start of treatment until disease progression. Stable disease (SD) was measured from the start of the treatment until criteria for disease progression were met. Time to progression was calculated from the first day of treatment until progression, death from any cause or last patient contact was to be known progression free or alive. In patients undergoing surgery after experiencing sufficient tumour shrinkage, any relapse, new occurrence of colorectal cancer or death was treated as events. Time to treatment failure (TTF) was calculated from the first day of treatment to the first occurrence of premature withdrawal due to adverse events, insufficient therapeutic response, death, failure to report, refusal of treatment, uncooperativeness or withdrawal of consent. OS was calculated from the first day of treatment to the date of death from any cause or the last date the patient was known to be alive.

### Treatment

The treatment schedule was: FOLFOX6 (oxaliplatin 100 mg m^−2^ on day 1, folinic acid 200 mg m^−2^ on day 1 and 5-fluorouracil 400 mg m^−2^ by 2-h i.v. bolus on day 1, followed by 3000 mg m^−2^ by 46-h continuous infusion starting on day 1) every 2 weeks for six cycles and bevacizumab 5 mg kg^−1^ on day 1 every 2 weeks for five cycles.

After the first 3 months of treatment, patients were evaluated for resectability: surgery was performed at least 8 weeks after the last dose of bevacizumab and 4 weeks after the last dose of chemotherapy. Resection had to be complete and macroscopically curative (R0). Radical surgery was defined as a margin of at least 1 mm. Other surgical treatments permitted were a two-stage hepatectomy; portal vein occlusion before major liver resection; and combined approach of radiofrequency (RFA) and hepatic resection. Patients undergoing intraoperative RFA were considered among those patients treated with radical intent. All patients treated with intraoperative RFA had lesions <3 cm.

Patients with non-resectable PR or SD after the first 6 cycles received 6 additional cycles of FOLFOX6+bevacizumab and were then re-evaluated for surgery.

After surgery, six additional courses of FOLFOX6+bevacizumab were administered to those patients operated on after receiving only six courses of therapy. This treatment was started 4–8 weeks after surgery, following functional evaluation of liver reserve and healing of surgical wounds. Postsurgical abdominal abscesses were considered major postsurgical complications, whereas wound-healing delays were treated as minor complications. Patients were evaluated by a multidisciplinary team composed of an oncologist, radiologist and surgeon at diagnosis, and again after 6 and 12 treatment cycles.

### Toxicity and dose modifications

The intensity of clinical adverse events was graded according to the NCI-CTC Version 3.0. In case of serious bevacizumab-related toxicity, bevacizumab treatment was temporarily suspended. Patients were excluded from receiving further treatment with bevacizumab, if they developed at least one of the following attributable toxicities: gastrointestinal perforation, arterial thromboembolic events, grade 3/4 haemorrhagic events, symptomatic grade 4 thrombosis, grade 4 hypertension or grade 4 proteinuria. If these were the only safety-related toxicities events, patients continued FOLFOX6 therapy on the planned schedule.

If a toxicity (haematological or gastrointestinal) was considered to be primarily attributable to chemotherapy and occurred at grade⩾2, chemotherapy was discontinued until toxicity resolved to grade 0–1 before restarting. If toxicity required a dosing delay of all study drugs for more than 3 weeks, the patient was withdrawn from the study for toxicity.

If paraesthesia with pain or persistent functional impairment were the only toxicities present at the next planned oxaliplatin administration, only oxaliplatin treatment was delayed, whereas administration of folinic acid, 5-fluorouracil and bevacizumab could continue on schedule. If the neurological toxicity was still present at the time of the next planned treatment cycle, oxaliplatin was discontinued permanently. Folinic acid, 5-fluorouracil and bevacizumab were then continued at the discretion of the investigator.

## Results

### Patients

Between September 2005 and July 2009, 21 patients were enrolled. On the basis of slow accrual of patients, we closed enrolment at 21 patients (16 in the first stage and 5 in the second stage). All of these patients had metastases only to the liver. The median size of liver metastases was 6 cm in longest diameter (range: 1–9.7 cm). Twenty patients received six biweekly cycles of FOLFOX6 and five cycles of bevacizumab, and were then evaluated for resectability. A PET-CT was performed both at baseline and within 1 month from the start of neoadjuvant therapy in 16 patients. Median time from last dose of chemotherapy to surgery was 8.8 weeks and from the last dose of bevacizumab to surgery was 10.8 weeks. Patient characteristics are summarised in Table 1.

### Toxicity

The main grade 3–4 toxicities reported were: neutropenia (19%), including one neutropenic fever resulting in death from sepsis, and thrombotic complications (14.3%). Haematological toxicity was likely due to chemotherapy, whereas the thrombotic side effects were due to bevacizumab. One patient discontinued FOLFOX6+bevacizumab after 4 cycles for gastrointestinal and haematological toxicity (grade 3). One patient received only one administration of bevacizumab because of a grade 3 thrombotic event and another received only four administrations due to a grade 1 haemorrhagic event.

Grade 1–2 and 3–4 adverse events according to NCI-CTC (version 3.0) are shown in Table 2.

### Evaluation of response

After the first 3 months of treatment, 12 patients (57.1%) experienced an OR (clinical CRs (cCRs): 3, 14.3% PR: 9, 42.8%), documented by RECIST criteria, and 20 patients had disease control (95.2%). Among radiological CRs, one patient achieved a radiological CR, but died from toxicity (sepsis in neutropenic fever) before surgery. One patient diagnosed with rectal cancer (12 cm from anal rima) and a single synchronous liver metastasis was evaluated as having SD by RECIST criteria, but analysis of the resected lesion did not reveal neoplastic cells. He had undergone surgery for the primary tumour, received medical treatment for 3 months and was then operated on for the liver metastasis.

### PET-CT analysis

Sixteen patients had a PET-CT scan before starting treatment and then again within 1 month of initiating treatment. Median SUVmax at baseline was 8. There were 11 patients with SUVmax ⩾8 at baseline. Among these 11 patients, we observed 2 cCRs (18 %), 5 PR (45.6%), 3 SD (27.4%) and 1 progressive disease (PD) (9%). Five patients had SUVmax values <8 at baseline. Among these, we observed one CR (20%), three PR (60%) and one SD (20%). Radical surgery was possible in 9 of the 11 patients (81.8%) who had basal SUVmax values ⩾8 (3 with CRs underwent biopsy to confirm pCR, 4 had PR, and 2 with SD underwent liver resection), whereas only 1 patient with basal SUVmax value <8 underwent surgery for CLM (*P*=0.909). Moreover after the first cycle of therapy, the median SUVmax was 5.2 (range: 0–8). Twelve patients had a reduction of SUVmax by at least 1 unit. The absolute value of variation was not detectable because it was not possible to measure SUVmax in all patients. Patients who experienced a SUVmax reduction had longer PFS (median PFS): 22 *vs* 14 months, *P*=0.0010). No correlation was found with OR or OS.

### Surgery and surgical complications—Outcome

Fourteen patients underwent surgery (66.6%): 13 had radical surgery (61.9%), whereas 1 patient underwent only explorative laparoscopy with no resection of the major liver involvement (4.7%). Twelve patients were operated on after six cycles and one patient after 12 cycles. Median time from the end of neoadjuvant therapy (FOLFOX6) to surgery was 8.8 weeks. Of the 14 patients who underwent surgery, 8 underwent only surgery (57.1), whereas 4 had surgery after portal vein embolisation, 1 had surgery with RFA and 1 patient had surgery with both portal vein embolisation and RFA.

In three cases, we documented a pCR (23%) on histological examination. Six patients (46%) experienced postsurgical complications: three patients had abdominal abscesses (major: 23%) and three had a delay in wound healing (minor: 23%). No postoperative mortality was recorded (defined as death within 60 days of surgery). Median time from surgery to commencement of systemic postoperative treatment was 43 days (range: 36–73 days).

No correlation was found between response rate and resection rate (*P*=0.369), OS and number of liver metastases (⩽3 *vs* > 3) (*P*=0.456), or between OS and response rate (*P*=0.404). The correlations with response in resected and non-resected patients are shown in Table 3.

**Figure 1:**
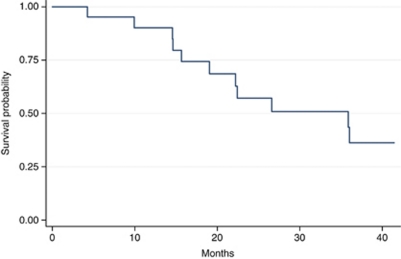
Overall survival.

**Figure 2:**
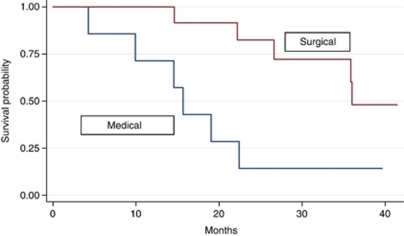
Overall survival in surgical and medical patients.

**Figure 3:**
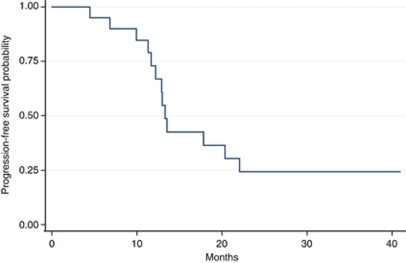
PFS.

As of the November 2009, the median follow-up was 3.2 years. Median OS was 22.5 months (range: 4.2–41.4 months) (Figure 1). Median OS in surgical compared with medical patients was 35.6 *vs* 15.3 months, respectively (*P*=0.009) (Figure 2). Median PFS was 12.9 months (range: 3.7–40.9 months) (Figure 3). Of the 14 resected patients, 7 relapsed (50%). Median disease-free survival (time from radical surgery to relapse) was 8 months (range: 5.9–13.5 months). Eleven patients died: 10 from disease progression and 1 from toxicity (sepsis in neutropenic fever). All results are reported in Table 4.

## Discussion

Neoadjuvant chemotherapy has been shown to downsize and increase the resection rate of unresectable CLM by 13–20% (Sharma *et al*, 2008). In the present study, we have shown that preoperative treatment with FOLFOX6, in association with bevacizumab, may convert 66.6% of patients with CLM from non-optimally resectable to resectable disease status. The original study was planned for 46 patients and we accrued only 21 patients over a 4-year period. Our decision to close recruitment before reaching the planned accrual was on the basis of difficulty in recruiting eligible patients. Therefore, all conclusions have been on the basis of smaller number of patients enrolled.

The best neoadjuvant regimen for downsizing CLM remains a matter of investigation: the present study suggests that the combination of FOLFOX6 with bevacizumab may be a very good option both in terms of efficacy (resectability rate: 66.6%) and major postoperative complications (23%). Moreover, the choice of the best biological agent is still controversial: a study by Folprecht *et al* (2010) showed that the use of cetuximab in combination with oxaliplatin- or irinotecan-based neoadjuvant chemotherapy resulted in a 28% increase in the resectability rate, with a stronger effect depending on K-ras status. On the contrary, the activity of bevacizumab is independent of K-ras status and, when used in combination with neoadjuvant chemotherapy, has obtained a high rate of pCRs, 23% in the present study and from 8 to 10% in recent reports (Ribero *et al*, 2007; Blazer *et al*, 2008; Gruenberger *et al*, 2008; Bouganim, 2009; Malavasi *et al*, 2009; Otsuka *et al*, 2009; Zorzi *et al*, 2010).

Only 23% of our patients presented major postsurgical complications in line with other reports, which also document that the use of bevacizumab in neoadjuvant setting did not affect complication rates when used with an interval of >5 weeks between the last bevacizumab administration and surgery (Benoist *et al*, 2006; Gruenberger *et al*, 2008; Kesmodel *et al*, 2008).

Our study did not demonstrate the correlation between response rate and the resection rate (*P*=0.369) reported by Folprecht *et al* (2005), or the pathologic response (Chun *et al*, 2009). This may be due to patient selection, that is, patients with initially ‘non-optimally resectable’, but potentially resectable metastases and patients whose tumours will never be resectable. In the first scenario, chemotherapy should offer a shrinkage of CLM, resulting in a higher resectability rate, whereas in the second scenario, chemotherapy has the role of extending disease control and maintaining quality of life. Moreover, we include in the definition of ‘non-optimally resectable patients’ not only anatomic/surgical criteria, but also criteria related to the correct timing of the surgical approach to liver metastases (i.e., synchronous liver metastases in patients with rectal cancer; 3 of 21 patients).

In addition, we investigated the role of PET-CT in detecting early responders to neoadjuvant treatment. Although the number of patients was small, the observed reduction in SUVmax (reduction of at least 1 unit) of FDG uptake does not seem to predict response (*P*=0.611) or resectability rate (*P*=0.909). However, patients who experienced a SUVmax reduction did have longer PFS (median PFS: 22 *vs* 14 months, *P*=0.0010). No correlation was found with OS.

In terms of outcome, there was no correlation between OS and the number of liver metastases (⩽3 *vs* > 3) (*P*=0.456), or between OS and response rate (*P*=0.404). Patients submitted to surgery survived longer: 15.3 *vs* 35.6 months (*P*=0.009). The present study confirms the importance of chemotherapy in association with surgery for CLM: for example, the results of a recent study by the European Organization for Research and Treatment of Cancer (EORTC, protocol 40983) suggests a 9.2% improvement in 3-year PFS in patients with potentially resectable liver disease who received perioperative chemotherapy as compared with surgery alone (Nordlinger *et al*, 2008).

In conclusion, administration of FOLFOX6+bevacizumab in this setting is feasible, does not increase the rate of major postsurgical complications, and therefore does not delay the resumption of chemotherapy after hepatic surgery. The correct definition of ‘resectability’ is still controversial, not only regarding anatomic/surgical criteria, but also regarding definition of the correct timing of primary tumour and hepatic surgery.

Early changes in PET-CT, in particular, reduction of SUVmax, seem to be a predictor of longer PFS, though the role of PET-CT should be validated in a wider setting.

## Figures and Tables

**Table 1:** Patient characteristics

	**(*N*=21)**	**%**
Median age, years: 61 (range, 37–72)		
		
*Sex*		
Male	16	76
Female	5	24
		
*ECOG*		
0	20	95
1	1	5
		
*TN clinical stage*		
T1N0	1	4, 8
T2N0	1	4, 8
T3N0	3	14, 3
T3N1	6	28, 6
T3N2	5	23, 8
T4N1	4	19
		
*No. of liver metastases*		
1–3	9	42, 9
4–5	1	4, 7
>5	11	52, 4
		
*CEA (ng* *ml*^−1^*)*	*N*=20	
⩽5	5	25
>5	15	75
		
*CA 19, 9 (U* *ml*^−1^*)*	*N*=20	
⩽37	7	35
>37	13	65

**Table 2:** Toxicity (first 6 cycles)

**Toxicity (*n*= 21)**	**G 1–2 (%)**	**G 3–4 (%)**	**Total (%)**
*Hematological toxicity*			
Neutropenia	4 (19)	4 (19)	8 (38)
Febrile neutropenia	0 (0)	1 (4.7)	1 (4.7)
Thrombocytopenia	1 (4.7)	0 (0)	1 (4.7)
			
*Gastrointestinal toxicity*			
Diarrhea	4 (19)	1 (4.7)	5 (23.7)
Nausea/vomiting	3 (14.3)	1 (4.7)	4 (19)
Mucositis	2 (9.5)	0 (0)	2 (9.5)
Infection of central venous catheter	0 (0)	1 (4.7)	1 (4.7)
Hypertension	1 (4.7)	0 (0)	1 (4.7)
Proteinuria	0 (0)	0 (0)	0 (0)
Thrombosis	0 (0)	3 (14.3)	3 (14.3)
Rectal bleeding	1 (4.7)	0 (0)	1 (4.7)
Paresthesia	2 (9.5)	0 (0)	2 (9.5)

**Table 3:** Objective response and surgery

	**Resected (%)**	**Not resected (%)**
*N* (on 21)	13 (61.9)	8 (38.1)
CR	2 (15.4)	1 (12.5)
PR	5 (38.5)	4 (50)
SD	6 (46.1)	2 (25)
PD	0 (0)	1 (12.5)

Abbreviations: CR=complete response; PD=progressive disease; PR=partial response; SD=stable disease.

**Table 4:** Results

	***N***	**%**
Objective response	12/21	57.1
R0 resection	13/21	61.9
Major postoperative complications	3/14	23
		
	**Median (months)**	**Range (months)**
Time to progression	12.1	3.1–39.7
Time to treatment failure	11.9	1.1–36.1
Progression-free survival	12.9	3.7–40.9
Overall survival	22.5	4.2–41.4
